# Alleviating the Effects of Short QT Syndrome Type 3 by Allele-Specific Suppression of the *KCNJ2* Mutant Allele

**DOI:** 10.3390/ijms252413351

**Published:** 2024-12-12

**Authors:** Ronald Wilders

**Affiliations:** Department of Medical Biology, Amsterdam Cardiovascular Sciences, Amsterdam University Medical Center, University of Amsterdam, 1105 AZ Amsterdam, The Netherlands; r.wilders@amsterdamumc.nl

**Keywords:** heart, human, ventricle, short QT syndrome, *KCNJ2*, Kir2.1, inward rectifier potassium current, mutations, cardiac cellular electrophysiology, computer simulations

## Abstract

Short QT syndrome type 3 (SQTS3 or SQT3), which is associated with life-threatening cardiac arrhythmias, is caused by heterozygous gain-of-function mutations in the *KCNJ2* gene. This gene encodes the pore-forming α-subunit of the ion channel that carries the cardiac inward rectifier potassium current (I_K1_). These gain-of-function mutations either increase the amplitude of I_K1_ or attenuate its rectification. The aim of the present in silico study is to test to which extent allele-specific suppression of the *KCNJ2* mutant allele can alleviate the effects of SQT3, as recently demonstrated in in vitro studies on specific heterozygous mutations associated with long QT syndrome type 1 and 2 and short QT syndrome type 1. To this end, simulations were carried out with the two most recent comprehensive models of a single human ventricular cardiomyocyte. These simulations showed that suppression of the mutant allele can, at least partially, counteract the effects of the mutation on I_K1_ and restore the action potential duration for each of the four SQT3 mutations that are known by now. We conclude that allele-specific suppression of the *KCNJ2* mutant allele is a promising technique in the treatment of SQT3 that should be evaluated in in vitro and in vivo studies.

## 1. Introduction

Short QT syndrome (SQTS), associated with a remarkably short heart rate-corrected QT interval (QTc interval) on the electrocardiogram (ECG) and first described in 2000 [1], is a rare inheritable and potentially life-threatening disease [2]. Only 220 patients (145 male) from 144 families were described in English-language medical journals from 2000 to 2017 [3]. There is no full consensus on the diagnostic criteria, including the cutoff value that should be used for a short QT or QTc interval [4,5,6,7]. Of note, several studies advocated that a short QTc interval ≤ 340 ms per se, as mentioned in the clinical guidelines of the European Society of Cardiology (ESC) [4], should not be used as the sole criterion for SQTS [8,9,10]. Moreover, such a short QTc interval ≤ 340 ms had a prevalence of ≈0.06% in the general population of Italy and “does not imply a significant risk of sudden death” [11]. However, in the study by Iribarren et al. [12] of the U.S. population, this prevalence was ≈0.5%, and a QTc interval ≤ 300 ms “was extraordinarily rare and was associated with significant ECG abnormalities and reduced survival”. In contrast, in the Japanese population, only 0.01% (2/19,153) of the subjects showed a QTc interval ≤ 350 ms [13].

Short QT syndrome type 3 (SQTS3 or SQT3; see Hancox et al. [14] for a comprehensible review of SQTS types 1–3) is caused by heterozygous gain-of-function mutations in the *KCNJ2* gene. This gene encodes the Kir2.1 pore-forming α-subunit of the ion channel that carries the cardiac inward rectifier potassium current (I_K1_) [15]. This membrane current is named after its inwardly rectifying characteristics in the non-physiological membrane potential range, which is more negative than its reversal potential (i.e., the Nernst potential for K^+^). In this membrane potential range, its ion flow becomes inward, and its current–voltage relationship “rectifies”. In the physiological membrane potential range, I_K1_ is an outward current that is strongly expressed in cardiac ventricular myocytes, dominates the final phase of their action potential, stabilizes their resting membrane potential, and is thus an important determinant of cardiac excitability (see Hibino et al. [16] and Gutiérrez et al. [17] for comprehensive reviews on I_K1_ and its function in health and disease). The gain-of-function mutations in *KCNJ2* either increase the amplitude of I_K1_ or attenuate its rectification, the latter meaning that its current–voltage relationship becomes more linear—or, more accurately, becomes less non-linear—in the physiological membrane potential range, as will be illustrated below. In either case, the outward current during the repolarization phase of the ventricular action potential (AP) is significantly increased, which shortens the AP duration as well as the associated refractory period and the QT and QTc intervals.

**Figure 1:**
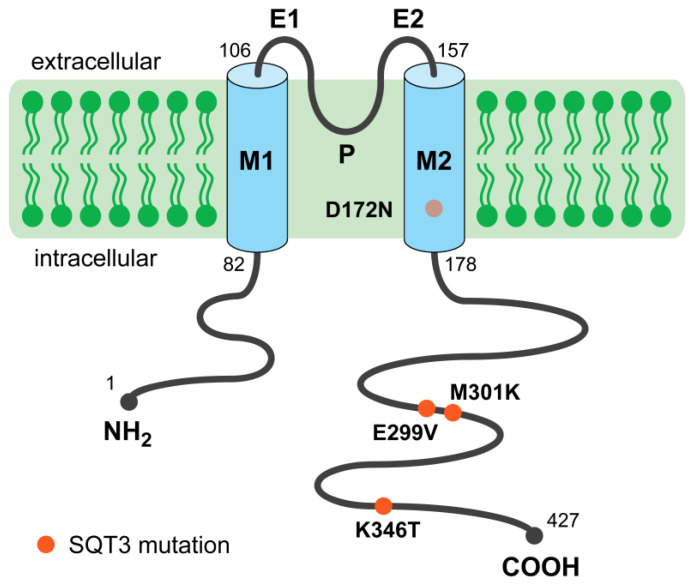
General topology of a Kir2.1 subunit with each of the four gain-of-function mutations that have been associated with the SQT3 syndrome. Each subunit consists of two membrane-spanning α-helical segments (M1 and M2) separated by the pore-forming domain P. The amino and carboxyl termini (NH_2_ and COOH, respectively) are located intracellularly. E1 and E2 indicate the two extracellular loops. The D172N mutation is located on the M2 inner helix. Four Kir2.1 α-subunits oligomerize to form a functional Kir2.1 potassium channel.

Thus far, four SQT3 mutations have been identified. Each of these SQT3 mutations is heterozygous: each of the SQT3 patients carries two different versions of the *KCNJ2* gene and is thus expected to produce approximately equal amounts of the regular “wild-type” and mutant Kir2.1 proteins. To date, no patients have been identified as homozygous, carrying two identical mutated versions of the *KCNJ2* gene, or compound heterozygous, carrying two different mutated versions of the *KCNJ2* gene. Ordered by their location on the Kir2.1 protein (Figure 1), the SQT3 mutations are D172N (p.Asp172Asn) [18], E299V (p.Glu299Val) [19], M301K (p.Met301Lys) [20], and K346T (p.Lys346Thr) [21]. Of note, the aspartic acid (Asp) at position 172, the glutamic acid (Glu) at position 299, and the methionine (Met) at position 301 all appear to be determinants of the inward rectification of the Kir2.1 channel [16]. The clinical observations for each of the four SQT3 mutations are summarized in Table 1, whereas data on the electrophysiological effects of each mutation are listed in Table 2. Three of the four mutations, i.e., D172N, E299V, and M301K, are regarded as definitively deleterious, making SQT3 the most pathogenic subtype of short QT syndrome [22].

**Table 1:** Mutations in *KCNJ2* observed in SQT3 patients and associated clinical observations.

Mutation	Location	Clinical Observations	Study
D172N	Transmembrane helix M2	QTc interval of 315 ms observed in an asymptomatic 5-year-old girl; narrow and peaked T waves. Ventricular fibrillation inducible by programmed electrical stimulation. QTc interval of 320 ms observed in the father, with a history of presyncopal events and palpitations since age 15. Genetic analysis revealed a novel (D172N) heterozygous mutation in the proband and suggested that this is a de novo mutation in the father.	Priori et al. [18]
E299V	C-terminus	Extremely short QTc interval (200 ms) observed in an 11-year-old boy. QRS complex merged with the T wave in the absence of a distinctive ST segment in all ECG leads. No ventricular arrhythmias on Holter or during exercise stress test, but episodes of paroxysmal atrial fibrillation observed on Holter. Genetic analysis demonstrated a novel (E299V) heterozygous mutation in the proband. The parents appeared negative for the mutation.	Deo et al. [19]
M301K	C-terminus	Extremely short QTc interval (194 ms) observed in an 8-year-old girl who showed multiple cardiac and non-cardiac disorders, including paroxysmal atrial fibrillation, inducibility of ventricular fibrillation, severe mental retardation, and epilepsy. Genetic analysis revealed a novel (M301K) heterozygous mutation in the proband. Family members displayed normal QTc intervals, but genetic data could not be obtained due to a lack of informed consent.	Hattori et al. [20]
K346T	C-terminus	Short QTc interval (331 ms) as well as narrow and peaked T waves observed in two male 9-year-old identical twins, also showing epilepsy and severe signs and symptoms of autism spectrum disorder. Novel heterozygous variant in *KCNJ2* identified, which was also found in the mother. When 8-year-old, a novel heterozygous variant in *KCNJ10*, encoding the Kir4.1 protein and also inherited from the mother, was reported in relation to the neurological findings.	Ambrosini et al. [21] Sicca et al. [23]

**Figure 2:**
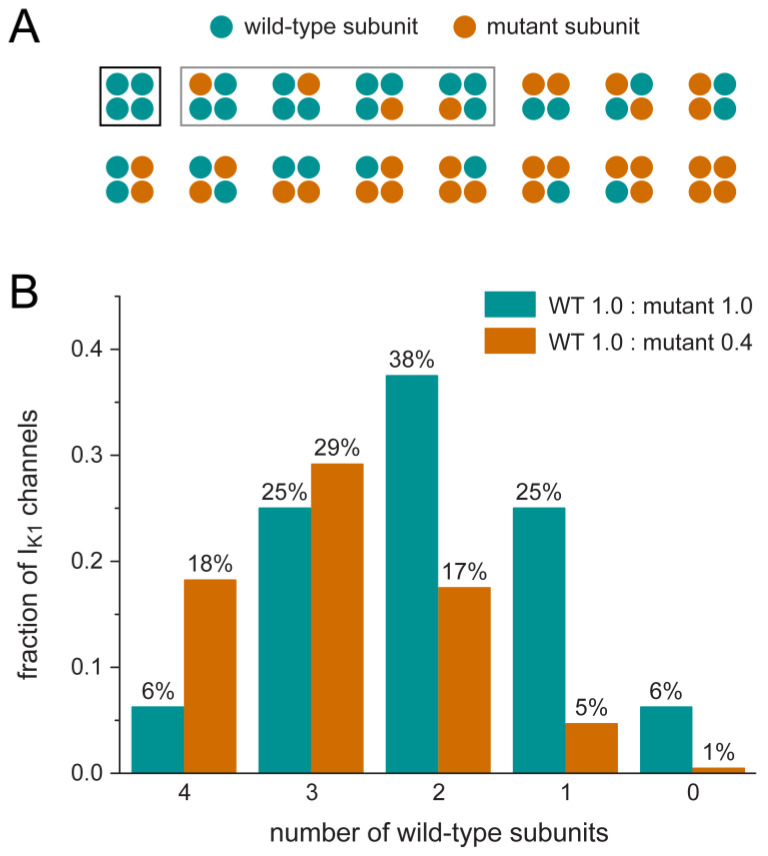
(**A**) Potential configurations of the tetrameric Kir2.1 channel in the case of a heterozygous mutation in *KCNJ2*. The rectangles indicate the five configurations with at most one mutant subunit (grey for exactly one mutant subunit; black for wild-type subunits only). (**B**) Binomial distribution of I_K1_ channels with 0–4 mutant subunits. Without suppression of the mutant allele, the distribution is symmetrical (cyan bars), and the fractions add up to 100%. With a 60% suppression of the mutant allele, the distribution is skewed (orange bars), and the fractions add up to 70% to account for the reduced channel density upon suppression and to allow a direct comparison of the amount of channels in each configuration.

Wild-type (WT) and mutant Kir2.1 subunits form homo- or heterotetramers that constitute the I_K1_ channel [24,25,26]. Because of the tetrameric structure of the Kir2.1 channel, only a minority of the channels will consist of purely WT subunits, provided that WT and mutant Kir2.1 subunits are translated and processed similarly and then randomly co-assemble into functional tetramers [27], as they do in the case of most loss-of-function (rather than gain-of-function) mutations in *KCNJ2* that are associated with Andersen–Tawil syndrome type 1, which is also known as long QT syndrome type 7 [28,29,30]. As a consequence, a mutation in *KCNJ2* may exert autosomal dominant effects because only 1/16th of the channels will contain four normally functioning WT subunits, whereas the remaining 15/16th of the channels will contain one to four mutant subunits (Figure 2). The same holds for the tetrameric channels that carry the cardiac rapid and slow delayed rectifier potassium currents (I_Kr_ and I_Ks_, respectively) and are encoded by the *KCNH2* and *KCNQ1* genes, respectively, so that heterozygous mutations in these genes may also exert a dominant negative effect upon random co-assembly into functional tetramers [31,32,33].

**Table 2:** Electrophysiological effects of the heterozygous mutations in *KCNJ2* associated with SQT3.

Mutation	Experimental Observations	Study
D172N	Significant ≈ 2-fold increase in I_K1_ amplitude at −65 and −55 mV (WT/D172N vs. WT), without changes in reversal potential, observed in voltage clamp experiments, carried out at room temperature, on CHO cells expressing Kir2.1 WT, D172N, or WT/D172N channels.	Priori et al. [18]
D172N	Significant ≈ 2.2-fold increase in I_K1_ amplitude at voltages between −70 and −40 mV (WT/D172N vs. WT), without significant changes in reversal potential, observed in ventricular action potential clamp experiments, carried out at 37 °C on CHO cells expressing Kir2.1 WT, D172N, or WT/D172N channels.	El Harchi et al. [34]
D172N	Significantly shortened action potentials with accelerated repolarization (WT/D172N vs. WT), but no significant hyperpolarization of resting potential, observed in dynamic clamp experiments carried out at 37 ± 1 °C on adult guinea pig left ventricular myocytes. Action potential duration at 90% repolarization (APD_90_) shortened by 27.7 ± 2.8% (mean ± SEM).	Du et al. [35]
E299V	Current density significantly decreased at potentials between −120 and −90 mV, but rectification strongly attenuated, without changes in reversal potential, in experiments carried out at room temperature on HEK-293 cells expressing Kir2.1 WT, E299V, or WT/E299V channels. I_K1_ amplitude significantly increased at voltages between −55 and +25 mV (WT/E299V vs. WT). Large outward current during early repolarization revealed in action potential clamp experiments.	Deo et al. [19]
M301K	Current density significantly decreased at potentials between −120 and −90 mV, but significantly increased at potentials between −30 and +100 mV (WT/M301K vs. WT) in experiments carried out at 37 °C on HEK-293 cells expressing Kir2.1 WT, M301K, or WT/M301K channels. Marked action potential abbreviation upon WT/M301K overexpression in neonatal rat ventricular myocytes in comparison with WT overexpression.	Hattori et al. [20]
K346T	Small but significant ≈19% increase in I_K1_ at −100 mV (WT/K346T vs. WT) in two-electrode voltage clamp experiments on *Xenopus laevis* oocytes carried out at room temperature. Increase in I_K1_ density likely due to increased surface expression of I_K1_ channels.	Ambrosini et al. [21]

CHO cells: Chinese hamster ovary cells; HEK-293 cells: human embryonic kidney cells.

It has been shown that allele-specific RNA interference with short-hairpin RNAs (shRNAs) to knock-down the mutant allele can largely restore the action potential duration (APD) of human-induced pluripotent stem cell-derived cardiomyocytes (hiPSC-CMs) and of ventricular cardiomyocytes isolated from transgenic rabbits in the case of (heterozygous) mutations in the *KCNH2* and *KCNQ1* genes [36,37,38,39]. It was also shown that allele-specific shRNAs reduced the incidence of arrhythmic events in hiPSC-CMs in the case of a pathogenic (heterozygous) long QT syndrome type 1 mutation in *KCNQ1* [40]. In the present in silico study, the two latest comprehensive models of a single human ventricular cardiomyocyte were used to assess the extent to which allele-specific suppression of the *KCNJ2* mutant allele may alleviate the effects of each of the aforementioned SQT3 mutations. The effect of each of the mutations was based on the in vitro data summarized in Table 2.

## 2. Results

### 2.1. Ventricular Cell Models and Incorporation of the SQT3 Mutations

**Figure 3:**
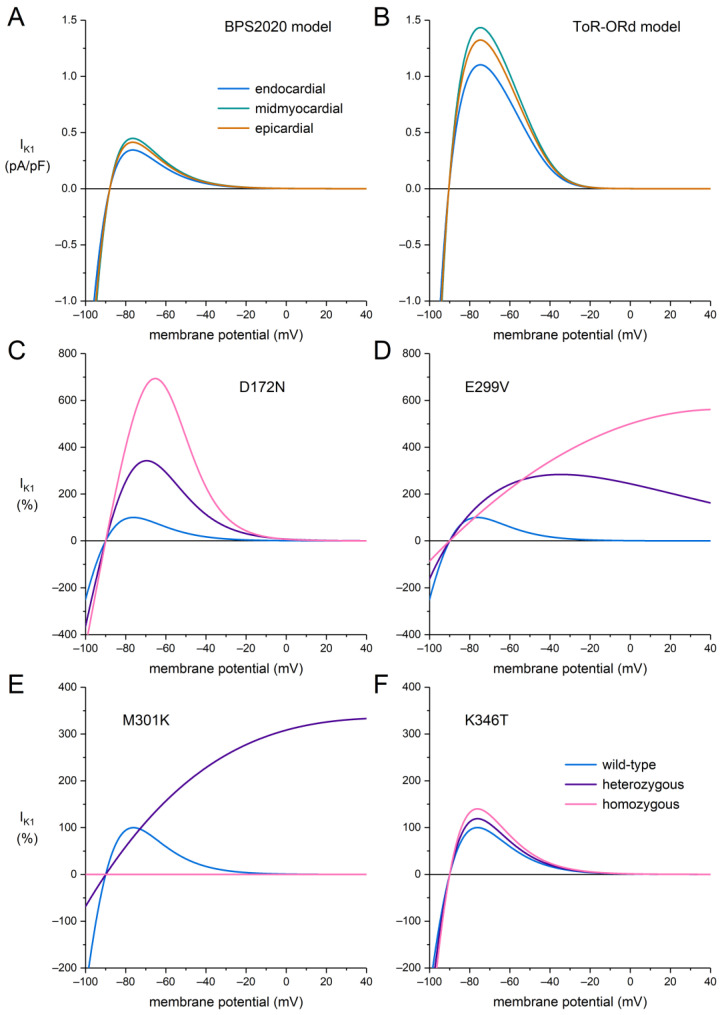
(**A**,**B**) I_K1_ current–voltage relationships of the BPS2020 and ToR–ORd models. (**C**–**F**) Effects of each of the SQT3 mutations in *KCNJ2* relative to the wild-type current. The maximum outward amplitude of the WT current is set to 100%. Note the difference in the ordinate scales of panels (**C**,**D**) on the one hand and panels (**E**,**F**) on the other hand.

We simulated the electrophysiology of an isolated human ventricular cardiomyocyte using the two latest comprehensive models of such a cell that were recently developed in parallel. These models were published in 2019 and 2020, and both can be considered major updates of the well-known and widely used O’Hara–Rudy dynamic (ORd) human ventricular cell model [41], which had become “the “gold standard” for in silico human ventricular cellular electrophysiology” [42]. Thus far, these two models are the only two major updates of the ORd model. The model published in 2019 is the “Tomek, Rodriguez—following ORd” model (“ToR–ORd model”) [43], and the one published shortly thereafter is the “Bartolucci–Passini–Severi, BPS2020” model [42]. Both models represent an endocardial myocyte by default but also have midmyocardial and epicardial versions, in which the density of I_K1_ is 30% and 20% larger, respectively. As illustrated in Figure 3A,B, the shape of the current–voltage relationship of I_K1_ of the two models is highly similar, but with a remarkable difference between the two models in their I_K1_ density, which is ≈3.2 times larger in the ToR–ORd model.

To incorporate the effects of the SQT3 mutations, the current–voltage relationships of the WT I_K1_ of the two models were both replaced with the “control I_K1_” presented in the supplement of the paper by Deo et al. [19] while retaining the aforementioned difference in current density. The shape of this control I_K1_ is highly similar to each of the shapes presented in Figure 3A,B, as illustrated in Section 4. The heterozygous and homozygous D172N and E299V mutations in *KCNJ2* were then implemented with the use of the equations that Deo et al. [19] fitted to the experimental data to reproduce the I_K1_ characteristics in each of these mutations (Figure 3C,D), as set out in Section 4.2 and Section 4.3 We used a similar approach to fit the experimental data that Hattori et al. [20] obtained on the M301K equation, as also set out in Section 4.2 and Section 4.3. In Figure 3E, the amplitude of the heterozygous M301K I_K1_ was scaled to the wild-type I_K1_ that was also recorded by Hattori et al. [20]. Because, intriguingly, Hattori et al. [20] recorded no functional I_K1_ in the homozygous case, the homozygous I_K1_ was set to zero in Figure 3E. The heterozygous and homozygous K346T current–voltage relationships of Figure 3F were obtained by increasing the wild-type current density by 19% and 40%, respectively, as observed by Ambrosini et al. [21] in their two-electrode voltage clamp experiments on *Xenopus laevis* oocytes and presented in their Figure 2 (see also Table 2).

In our action potential simulations, we used the heterozygous I_K1_ equations now that each of the four SQT3 mutations is heterozygous in the clinic (Table 1). It is important to realize that it is to be expected that the effects of the mutations are larger in the ToR–ORd model than in the BPS2020 model (see Section 3.3). This is because the WT I_K1_ is substantially larger in the ToR–ORd model (Figure 3A,B), so changes in this current due to a mutation should have larger effects if not all other currents are also larger. In all simulations, I_K1_ was a voltage-dependent but time-independent current, as in both models.

As illustrated in Figure 2, a 60% suppression of the mutant allele results in a decrease in the number of I_K1_ channels to 70% of control and an increase in the number of purely WT channels (from 6% to 18% of control), which underlie the WT current–voltage relationship of Figure 3C–F. As a sort of an “educated guess”, we speculated that the remaining channels with 1–4 mutant subunits would show the heterozygous current–voltage relationships of Figure 3C–F. Thus, we simulated the current–voltage relationship of I_K1_ in the case of a heterozygous mutation in *KCNJ2* as a combination of 18% and 52% of the WT and heterozygous I_K1_, respectively, and termed this setting “suppression A”. However, given the substantially larger homozygous I_K1_ as compared to the heterozygous I_K1_ for each of the mutations, except for the intriguing M301K mutation, it is conceivable that a large fraction of the channels composed of both WT and mutant subunits, for example, those with only one mutant subunit, behave like WT channels. In the latter case, the 18% and 52% fractions would change to 47% and 23% (Figure 2), together still adding up to 70%. We also used these fractions in our simulations and termed this setting “suppression B”.

### 2.2. Effects of the D172N Mutation and Suppression of the Mutant Allele

**Figure 4:**
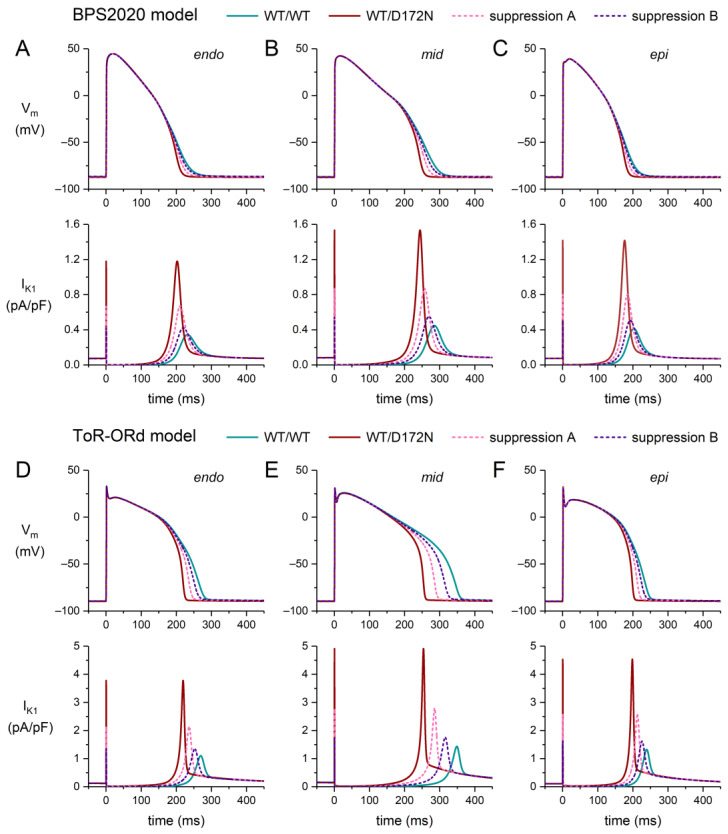
Effects of the heterozygous D172 mutation in *KCNJ2* (WT/D172N) on the action potential of a human ventricular cardiomyocyte, stimulated at 1 Hz, in the endocardial (‘endo’), midmyocardial (‘mid’), and epicardial (‘epi’) versions of the BPS2020 and ToR–ORd models, and effects of suppression of the mutant allele (dashed lines). (**A**–**C**) Endo (**left**), mid (**middle**), and epi (**right**) action potentials (**top**) and associated I_K1_ (**bottom**) in the BPS2020 model. (**D**–**F**) Endo (**left**), mid (**middle**), and epi (**right**) action potentials (**top**) and associated I_K1_ (**bottom**) in the ToR–ORd model. V_m_ denotes the membrane potential. Suppression settings “A” and “B” as set out in Section 2.1.

Figure 4 shows the effects of the heterozygous WT/D172 mutation in *KCNJ2* on the action potential and associated I_K1_ of a human ventricular cardiomyocyte in the endocardial (‘endo’), midmyocardial (‘mid’), and epicardial (‘epi’) versions of the BPS2020 model (Figure 4A–C) and the ToR–ORd model (Figure 4D–F). Because the effects of the mutation are largely restricted to an increase in the I_K1_ amplitude, without major changes in the shape of the curve–voltage relationship (Figure 3C), the effects of the mutation on the AP shape are observed during the AP repolarization phase, where I_K1_ is large, rather than the plateau phase, where I_K1_ is small. In both models, the largest AP shortening is observed in the mid version, with the highest I_K1_ amplitude (Figure 4A–F, bottom panels). In the mid version of the BPS2020 model, the AP shortening is limited to 35 ms (Figure 4B, top panel). In the ToR–ORd model, the AP shortening is substantially higher, which is to be expected from the higher I_K1_ amplitude in this model, and amounts to 91 ms (Figure 4E, top panel). Suppression A and B (see Section 2.1 for the associated simulation settings) reduce this shortening from 91 to 59 ms and 30 ms, respectively, thus largely, but not completely, restoring the AP duration.

### 2.3. Effects of the E299V Mutation and Suppression of the Mutant Allele

**Figure 5:**
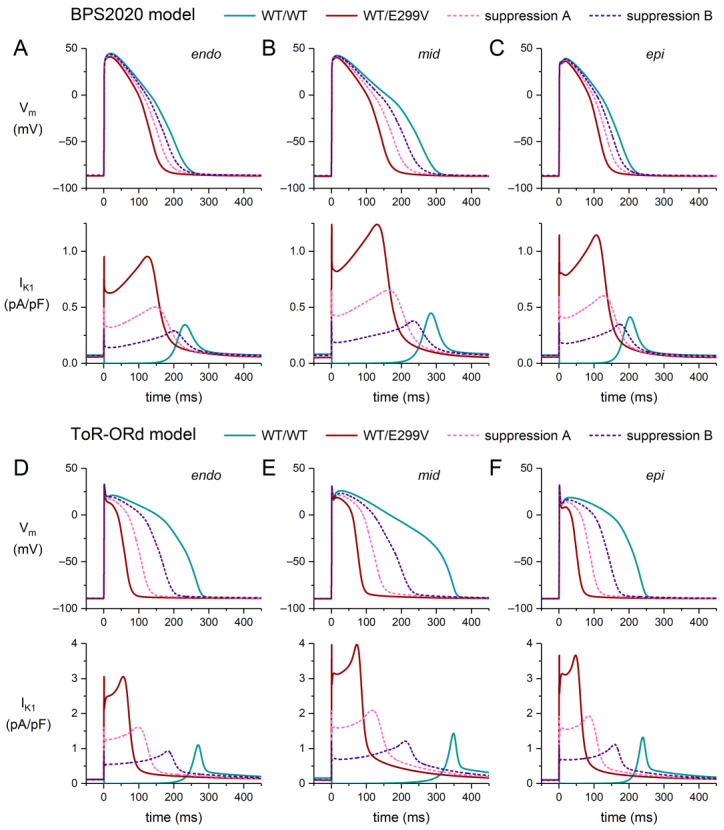
Effects of the heterozygous E299V mutation in *KCNJ2* (WT/E299V) on the action potential of a human ventricular cardiomyocyte, stimulated at 1 Hz, in the endocardial (‘endo’), midmyocardial (‘mid’), and epicardial (‘epi’) versions of the BPS2020 and ToR–ORd models, and effects of the suppression of the mutant allele (dashed lines). (**A**–**C**) Endo (**left**), mid (**middle**), and epi (**right**) action potentials (**top**) and associated I_K1_ (**bottom**) in the BPS2020 model. (**D**–**F**) Endo (**left**), mid (**middle**), and epi (**right**) action potentials (**top**) and associated I_K1_ (**bottom**) in the ToR–ORd model. Suppression settings “A” and “B” as set out in Section 2.1.

Figure 5 shows the effects of the heterozygous WT/E299V mutation in the same format as Figure 4. Unlike the D172 mutation, the E299V mutation strongly attenuates the rectification of I_K1_ (Figure 3D), resulting in a substantial outward current during the plateau phase of the action potential (Figure 5A–F, bottom panels) and an associated impressive AP shortening, in particular in the ToR–ORd model (Figure 5D–F, top panels). In the BPS2020 model, the AP shortening amounts to 71, 116, and 63 ms in the endo, mid, and epi versions, respectively (Figure 5A–C, top panels). In the ToR–ORd model, with its higher I_K1_ amplitude (Figure 5A–F, bottom panels), these numbers are as large as 193, 257, and 174 ms, respectively (Figure 5D–F, top panels). One may argue that the AP shortening and the associated shorter refractory period are potentially arrhythmogenic. On the other hand, however, the transmural dispersion of repolarization is also reduced, which may be considered anti-arrhythmogenic [44]. Suppression of the mutant allele by 60% reduces the I_K1_ amplitude (Figure 5A–F, bottom panels), but the remaining current is still able to shorten the AP plateau phase to a considerable extent (Figure 5A–F, top panels), resulting in an AP shortening of 80, 131, and 75 ms in the endo, mid, and epi versions of the ToR–ORd model, respectively, in the case of suppression B (Figure 5D–F, top panels).

### 2.4. Effects of the M301K Mutation and Suppression of the Mutant Allele

**Figure 6:**
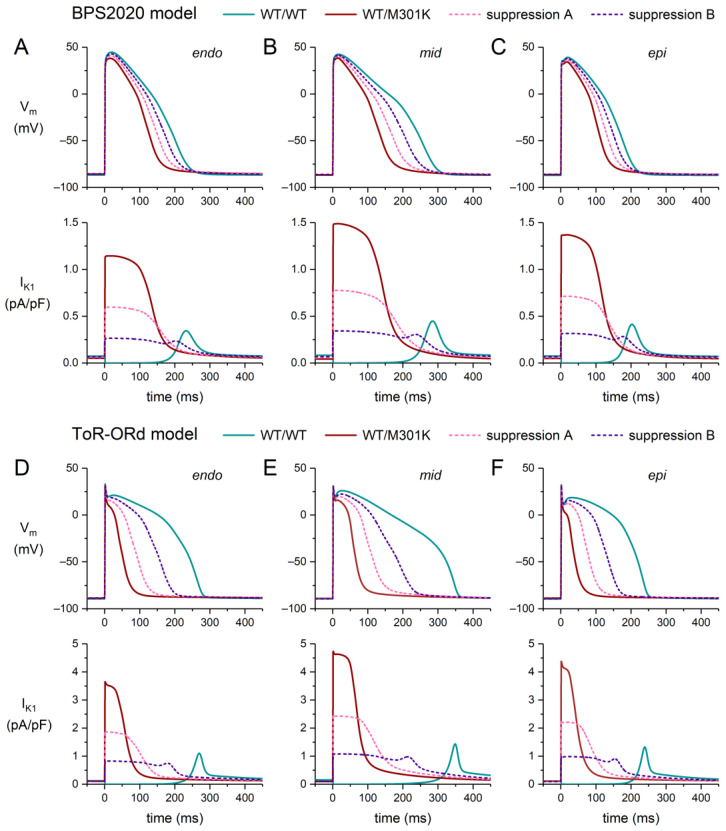
Effects of the heterozygous M301K mutation in *KCNJ2* (WT/M301K) on the action potential of a human ventricular cardiomyocyte, stimulated at 1 Hz, in the endocardial (‘endo’), midmyocardial (‘mid’), and epicardial (‘epi’) versions of the BPS2020 and ToR–ORd models, and effects of the suppression of the mutant allele (dashed lines). (**A**–**C**) Endo (**left**), mid (**middle**), and epi (**right**) action potentials (**top**) and associated I_K1_ (**bottom**) in the BPS2020 model. (**D**–**F**) Endo (**left**), mid (**middle**), and epi (**right**) action potentials (**top**) and associated I_K1_ (**bottom**) in the ToR–ORd model. Suppression settings “A” and “B” as set out in Section 2.1.

Like the E299V mutation, the M301K mutation strongly attenuates the rectification of I_K1_ (Figure 3E). In the plateau membrane potential range, the WT/M301K current is even larger than the WT/E299V current (Figure 3D,E). This results in a somewhat more pronounced abbreviation of the AP plateau and a stronger AP shortening (Figure 6A–F, top panels). In the endo, mid, and epi versions of the ToR–ORd model, the AP shortening now amounts to 196, 263, and 180 ms, respectively. As in the case of the E299V mutation, suppression of the mutant allele by 60% is not sufficient to restore the AP shortening, in particular in the case of the ToR–ORd model, due to the large outward current that remains during the AP plateau (Figure 6A–F, bottom panels).

### 2.5. Effects of the K346T Mutation and Suppression of the Mutant Allele

**Figure 7:**
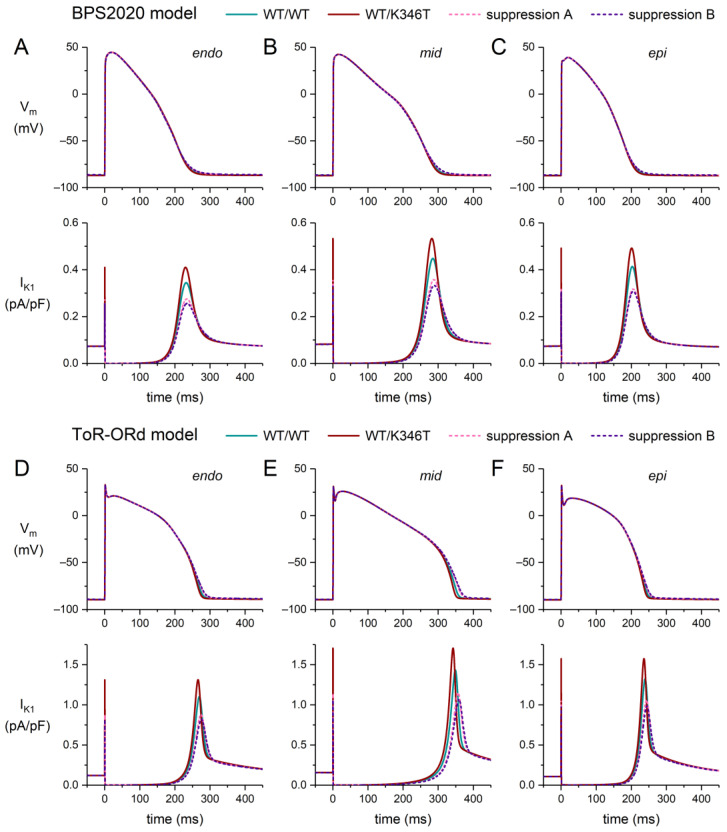
Effects of the heterozygous K346T mutation in *KCNJ2* (WT/K346T) on the action potential of a human ventricular cardiomyocyte, stimulated at 1 Hz, in the endocardial (‘endo’), midmyocardial (‘mid’), and epicardial (‘epi’) versions of the BPS2020 and ToR–ORd models, and effects of the suppression of the mutant allele (dashed lines). (**A**–**C**) Endo (**left**), mid (**middle**), and epi (**right**) action potentials (**top**) and associated I_K1_ (**bottom**) in the BPS2020 model. (**D**–**F**) Endo (**left**), mid (**middle**), and epi (**right**) action potentials (**top**) and associated I_K1_ (**bottom**) in the ToR–ORd model. Suppression settings “A” and “B” as set out in Section 2.1.

Unlike the E299V and M301K mutations, the K346T mutation does not attenuate the rectification of I_K1_ but increases its current density by 19% (Figure 3F). The effect of this mutation on I_K1_ is therefore qualitatively, but not quantitatively, comparable with the D172N mutation (Figure 3C,F). The increase in I_K1_ density is, however, so small that the resulting AP shortening is almost negligible, particularly in the case of the BPS2020 model (Figure 7). Suppression of the mutant allele reduces the I_K1_ density beyond control levels (Figure 7A–F, bottom panels, dashed lines). Consequently, the action potential is even prolonged beyond its WT level upon suppression of the mutant allele (Figure 7A–F, top panels, dashed lines).

### 2.6. Effects of the D172N and E299V Mutations on APD Restitution

The restitution of the AP duration (APD restitution), i.e., the adaptation of the action potential to a sudden change in stimulation rate, has been proposed as an important determinant of the occurrence of re-entrant arrhythmias, or at least the stability of such arrhythmias [45]. Therefore, we constructed APD restitution curves for two of the mutations, i.e., one of the two mutations that mainly act on the density of I_K1_ (D172N and K346T) and one of the two mutations that mainly act on its rectification (E299V and M301K). We selected E299V as being representative of both E299V and M301K, and we did not select K346T because of the minor effects of this mutation in our simulations (Figure 7). Also, we restricted our simulations to the default endo versions of the two models and their mid versions.

**Figure 8:**
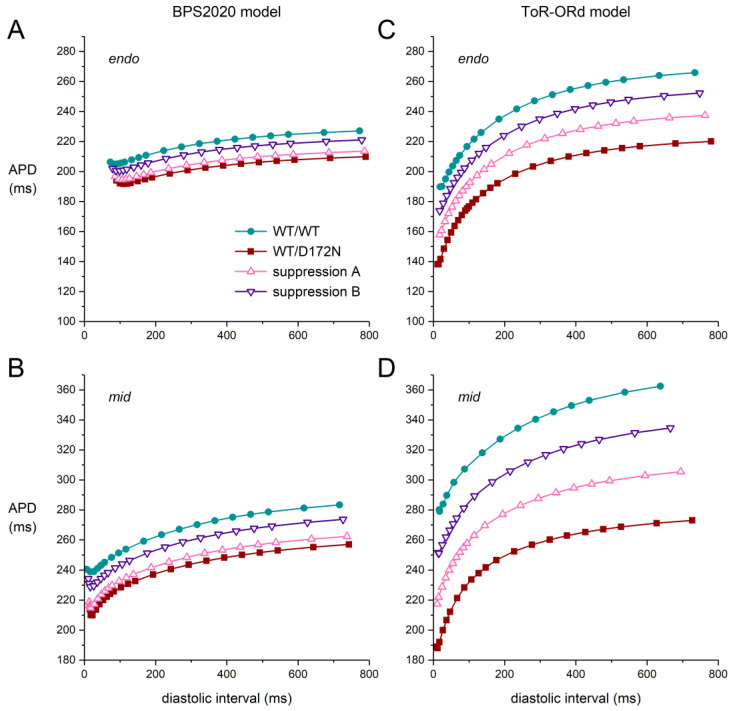
APD restitution curves in the case of the heterozygous D172N mutation obtained with an S1-S2 stimulus protocol. After a train of S1 stimuli at 1 Hz, an S2 stimulus was applied at various diastolic intervals, and the APD upon the S2 stimulus was determined. (**A**,**B**) APD restitution curves obtained with the (**A**) endo and (**B**) mid versions of the BPS2020 model. (**C**,**D**) APD restitution curves obtained with the (**C**) endo and (**D**) mid versions of the ToR–ORd model. To allow for a direct comparison, the APD range for the two endo models is identical, as is the APD range for the two mid models.

Figure 8 shows the APD restitution curves for the D172N mutation. A comparison of the WT/WT control APD restitution curves of the two models in Figure 8A,C immediately unveils a striking difference in their shape and steepness, which remains upon the implementation of the D172N mutation and upon the implementation of suppression of the mutant allele. The rate adaptation of the APD is much stronger in the ToR–ORd model than it is in the BPS2020 model. Furthermore, the APD restitution curves of the BPS2020 model are biphasic, with an increase in APD at short diastolic intervals. Such differences between cardiac cell models often remain more or less hidden but are not uncommon (see, e.g., Cherry et al. [46]).

The D172N mutation increases the slope of the APD restitution curves, which is partially restored upon suppression of the mutant allele. Now that it is generally agreed that steeper restitution is pro-arrhythmic (see Árpádffy-Lovas et al. [47] and primary references cited therein), this points to a pro-arrhythmic effect of the D172N mutation, which is reinforced by the concomitant AP shortening and the associated shortening of the refractory period. It is tempting to relate this finding to the inducibility of ventricular fibrillation in the proband (Table 1), but one should realize that the clinical data are limited to a single patient.

**Figure 9:**
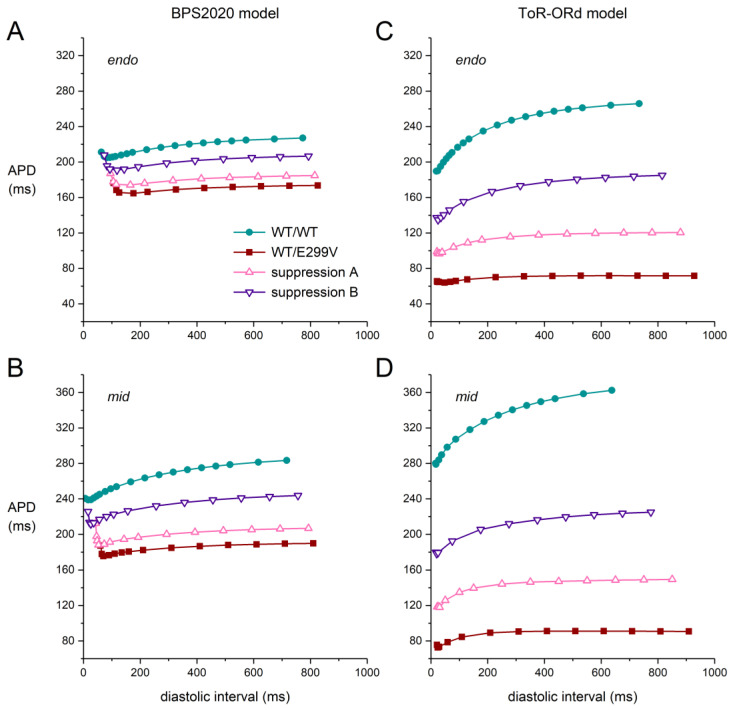
APD restitution curves in the case of the heterozygous E299V mutation obtained with an S1-S2 stimulus protocol. After a train of S1 stimuli at 1 Hz, an S2 stimulus was applied at various diastolic intervals, and the APD upon the S2 stimulus was determined. (**A**,**B**) APD restitution curves obtained with the (**A**) endo and (**B**) mid versions of the BPS2020 model. (**C**,**D**) APD restitution curves obtained with the (**C**) endo and (**D**) mid versions of the ToR–ORd model. To allow for a direct comparison, the APD range for the two endo models is identical, as is the APD range for the two mid models.

In the case of the E299V mutation, the APD restitution curves are flattened rather than steepened (Figure 9), pointing to an anti-arrhythmic effect of the E299V mutation. Accordingly, one might say no ventricular arrhythmias were observed during Holter recordings or during exercise stress tests (Table 1). However, one should again realize that the clinical data are limited to a single patient. Moreover, ventricular fibrillation was inducible in the single patient with the M301K mutation (Table 1), which has highly similar effects on I_K1_ (Figure 3).

## 3. Discussion

In the present study, we have demonstrated that suppression of the mutant allele can, at least partially, restore the action potential duration for each of the four SQT3 mutations that are known by now. Therefore, we conclude that allele-specific suppression of the *KCNJ2* mutant allele is a promising technique in the treatment of SQT3 that should be evaluated in in vitro and in vivo studies.

### 3.1. Gain-of-Function Mutations in Kir2.1

Interestingly, Xia et al. [48] reported a gain-of-function mutation in Kir2.1 (V93I (p.Val93Ile)) that was clearly associated with familial atrial fibrillation but showed no clinical effects on the ventricles, unlike the four gain-of-function mutation in Kir2.1 associated with SQT3. Yet, whole-cell voltage clamp experiments on COS-7 cells revealed increased homozygous and heterozygous V93I mutant Kir2.1 currents over the entire voltage range, similar to the current–voltage relationships of the SQT3-related D172N and K346T mutations (Figure 3C,F) and likely due to an increase in the single channel conductance of the Kir2.1 channels [48]. A V93I-induced increase was also observed in a comparison of homozygous Kir2.1 mutant current with wild-type current in voltage clamp experiments on transfected CHO-K1 cells by Zaklyazminskaya et al. [49]. However, they identified the V93I mutation in a 15-year-old proband and her father, who showed significant and borderline QTc prolongation, respectively, rather than a QTc shortening.

Recently, Moreno-Manuel et al. [50,51] studied the E299V and M301K mutations in mouse models generated by intravenous cardiac-specific adeno-associated virus-mediated gene transfer (using the AAV9 vector). The E299V mice showed extreme QT shortening and, accordingly, a dramatic decrease in the AP duration of isolated ventricular cardiomyocytes [51]. No changes were observed in their resting membrane potential and AP upstroke velocity. Voltage clamp experiments on these cardiomyocytes carried out at room temperature revealed an unchanged density of I_K1_ at potentials negative to −80 mV—at odds with the aforementioned observations by Deo et al. [19] (Table 2)—as well as an unchanged resting membrane potential, but a significantly increased I_K1_ at potentials positive to −50 mV as a result of a strongly attenuated rectification. Interestingly, and unexpectedly, the E299V ventricular cardiomyocytes showed significant hyperpolarizing shifts in the current–voltage relationship of the fast sodium current (I_Na_), which flows through Na_V_1.5 channels, as well as its steady-state activation and inactivation curves, without a change in the I_Na_ peak amplitude.

In the M301K model, the “Kir2.1^M301K^ mice” showed a significantly shortened QTc interval, a widened QRS complex, and inducible ventricular arrhythmias [50]. The I_K1_ density was decreased at potentials negative to −80 mV but increased at potentials positive to −50 mV as a result of a strongly attenuated rectification. As in the case of the E299V model, I_Na_ showed hyperpolarizing shifts in its current–voltage relationship as well as in its steady-state activation and inactivation curves. However, the I_Na_ peak amplitude showed a significant decrease, which is in line with the also observed significant increase in QRS duration. The decrease in I_Na_ peak amplitude could be associated with a decrease in Na_V_1.5 channels reaching the membrane. The similar decrease in the I_K1_ and I_Na_ densities can be explained by the combined trafficking of Kir2.1 and Na_V_1.5 from the sarcoplasmic reticulum to the cell membrane [52,53]. The mutually reduced density confirms the reciprocal modulation of I_K1_ and I_Na_ channels in macromolecular complexes [54]. However, in the particular case of the M301K mutation, the reduced density of I_K1_ is accompanied by an increase in functional I_K1_ due to the strongly attenuated rectification, so that the decrease in I_Na_ is accompanied by an increase in I_K1_ rather than a decrease, thus reducing ventricular excitability instead of preserving it [17].

### 3.2. Availability of Experimental Data

We had to base our simulations on a limited amount of experimental data, mostly obtained in Kir2.1 expression systems at room temperature (Table 2). Data obtained under more close-to-physiological conditions are not available. In our simulations, we used a 19% increase in I_K1_ density to model the WT/K346T mutation based on the experimental observations by Ambrosini et al. [21] on *Xenopus laevis* oocytes. However, in their supplemental data, they showed a substantially larger increase (by ≈53%), without changes in reversal potential or rectification properties, obtained in voltage clamp experiments on HEK-293 cells (WT/K346T vs. WT), also carried out at room temperature. So, it may well be that we have underestimated the effects of the K346T mutation in our simulations.

Kir2.1 is by far the most abundant Kir2 subunit in the ventricles of mammalian species, including humans [55]. However, one should not overlook that Kir2.2 and Kir2.3, encoded by the *KCNJ12* and *KCNJ4* genes, respectively, are also expressed (see Reilly and Eckhardt [15] and studies cited therein). Kir2.2 and Kir2.3 cannot only form homomeric channels with a current–voltage profile that differs from Kir2.1, but they can also form fully functional heteromeric channels with Kir2.1, with yet another current–voltage profile [56]. In the present study, the potential role of such “alternative” I_K1_ channels was not taken into account because almost all experimental data on the effects of mutations in Kir2.1 have been obtained in Kir2.1 expression systems.

### 3.3. Model Dependence of Simulation Results

**Figure 10:**
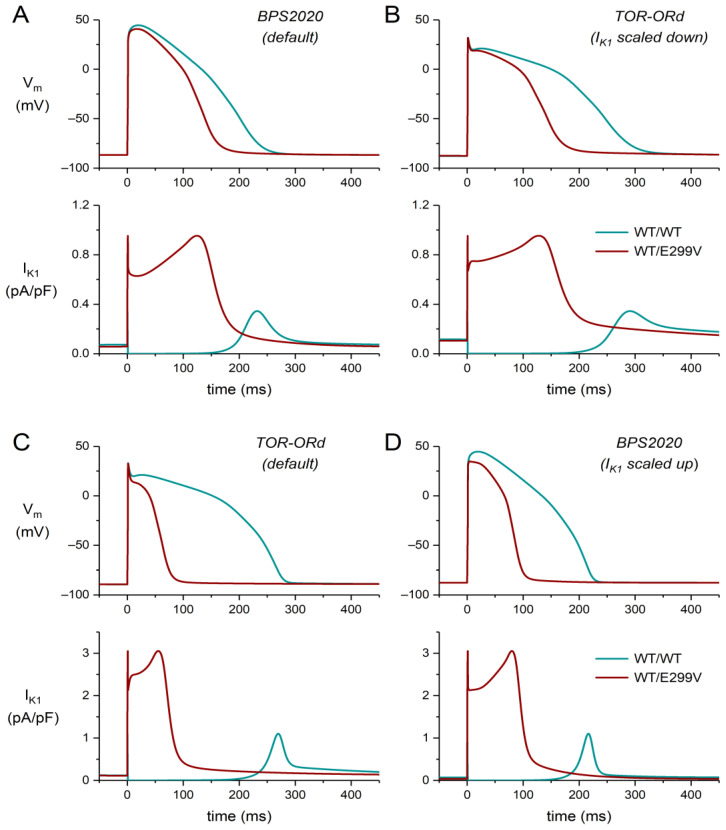
Effects of the heterozygous E299V mutation on the action potentials obtained during 1 Hz stimulation in simulations in which the I_K1_ densities of the BPS2020 and ToR–ORd models were matched by either scaling down the I_K1_ density of the ToR–ORd model by a factor of 3.2012 or scaling up the I_K1_ density of the BPS2020 model by this factor. (**A**) Membrane potential (V_m_; **top**) and I_K1_ (**bottom**) of the default BPS2020 model. (**B**) V_m_ and I_K1_ of the ToR–ORd model with its I_K1_ density scaled down. (**C**) V_m_ and I_K1_ of the default ToR–ORd model. (**D**) V_m_ and I_K1_ of the BPS2020 model with its I_K1_ density scaled up. Note the difference in the I_K1_ ordinate scales between panels (**A**,**B**) on the one hand and panels (**C**,**D**) on the other hand.

If anything, our simulations demonstrate that human ventricular cell models can produce quantitatively quite different results, even if they can both be considered major updates of the well-known and widely used O’Hara–Rudy dynamic (ORd) cell model and are largely based on the same set of currently available experimental data [42,43]. Part of the differences can be explained by the much larger I_K1_ density in the ToR–ORd model as compared to the BPS2020 model (Figure 3A,B). This striking difference in I_K1_ density between the two models results from differences in the role of I_K1_ in the optimization criteria that were used in the development of the two models (see Section 4.2 and Section 4.3). To assess the role of the formulation of I_K1_ in the quantitative differences in the simulation results between the two models, we ran simulations in which we swapped the I_K1_ densities of the models, which differ by a factor of 3.2012, as set out in Section 4.3. For this purpose, we ran simulations with the ToR–ORd model with its I_K1_ density scaled down by a factor of 3.2012, thus setting it equal to the I_K1_ density of the default BPS2020 model (Figure 10A,B). Similarly, we ran simulations with the BPS2020 model with its I_K1_ density scaled up by the same factor of 3.2012, setting it equal to the I_K1_ density of the default ToR–ORd model (Figure 10C,D). In these illustrative simulations, we evaluated the effect of the heterozygous E299V mutation on the action potentials obtained during 1 Hz stimulation. As shown in Figure 10, the differences between the data obtained with the two models are much smaller in this case than in Figure 5A,D. With the default BPS2020 model, the E299V mutation shortens the APD by 71 ms (31%; Figure 5A). However, with the default ToR–ORd model, the mutation-induced decrease in APD amounts to 193 ms (73%; Figure 5D). If the I_K1_ density in the ToR–ORd model is set to the same low level as in the BPS2020 model, the mutation-induced decrease in APD is 116 ms (41%), which is much closer to the 71 ms (31%) decrease observed with the default BPS2020 model (Figure 10A,B). Conversely, if the I_K1_ density in the BPS2020 model is set at the same high level as in the ToR–ORd model, the mutation-induced decrease in APD becomes 119 ms (55%), which is much closer to the 193 ms (73%) decrease observed with the default ToR–ORd model (Figure 10C,D). These results on the effects of differences in I_K1_ density between the models highlight the importance of knowing the exact I_K1_ density of human ventricular cardiomyocytes. However, experimental data from the literature show large variations in the amplitude of this I_K1_, as summarized by Meijer van Putten et al. [57] in their Figure 4.

Also, our simulations revealed substantial differences between the APD restitution curves of the two models (Figure 8) that should be taken into account when selecting a human ventricular cell model for in silico studies. Thus, our simulation results demonstrated a rather striking “model dependence” [58]. It has been shown that the two models exhibit more differences. Our Figure 4, Figure 5, Figure 6 and Figure 7 already reveal a remarkably different AP shape between the models, in particular with respect to the presence of a clear spike-and-dome morphology. Another remarkable difference is the large difference in the amplitude of the calcium transient, as illustrated by Sutanto and Heijman [58]. A thorough comparison of the electrophysiological behavior of the two models—beyond the differences in their I_K1_ densities and the effects of matching these densities, as in Figure 10—would be very useful. However, this is a challenging project that is well beyond the scope of the present study.

### 3.4. Allele-Specific Suppression

Allele-specific suppression of the mutant allele has been tested in several studies of heterozygous cardiac ion channelopathies. For example, Matsa et al. [36] demonstrated that allele-specific RNA interference can “normalize” the action potential duration (APD) of long QT syndrome type 2-associated hiPSC-CMs with the A561T (p.Ala561Thr) loss-of-function mutation in *KCNH2*, thus exhibiting a reduced I_Kr_. In a more recent study, Bains et al. [38] followed a “suppression-and-replacement” (“SupRep”) approach with shRNAs to restore the APD of hiPSC-CMs with loss-or-function or gain-of-function mutations in *KCNH2*, associated with long QT syndrome type 2 and short QT syndrome type 1 and thus exhibiting a reduced or an increased I_Kr_, respectively. With this technique, they could not only knock-down the target allele by ≈80% (suppression) but also introduce the expression of a healthy *KCNH2* replacement copy (replacement). Using this SupRep approach, they were able to fully restore the APD of their hiPSC-CMs with the G604S (p.Gly604Ser) or N633S (p.Asn633Ser) loss-of-function mutations in *KCNH2* associated with long QT syndrome type 2. Similarly, the SupRep technique was able to substantially increase the shortened APD of hiPSC-CMs carrying the N558K (p.Asn558Lys) gain-of-function mutation in *KCNH2* associated with short QT syndrome type 1, although not to the control level, as in our simulations of the SQT3 mutations. They had already applied this SupRep technique to hiPSC-CMs exhibiting an AP prolongation due a reduced I_Ks_ as a result of a loss-of-function mutation in the *KCNQ1* gene associated with long QT syndrome type 1 and demonstrated that it could substantially, but not fully, reduce the AP prolongation [37]. The potential of the SupRep gene transfer has not only been tested in in vitro studies. Very recently, the SupRep gene therapy was successfully applied to transgenic rabbits with type 1 long QT syndrome, where it reduced the mutation-induced QT prolongation as well as the AP duration of isolated ventricular cardiomyocytes [39].

Ultimately, allele-specific suppression, whether or not in combination with replacement, should find its way into the clinic. There are, however, several issues that need to be recognized, as reviewed by Greener and Donahue [59], Bongianino and Priori [60], and Zhang et al. [61]. (Bio)medical issues can be formulated as a number of questions. Can the construct of interest, whether it is complementary DNA (cDNA) for replacement or shRNA (or other interfering RNA) for suppression, be delivered in an efficient and non-immunogenic way? Will the potentially heterogeneous transduction of the targeted cardiomyocytes not result in a pro-arrhythmic heterogeneity in AP duration? Will the targeted system not react to the gene transfer by some compensatory response? Is the gene expression sufficiently stable over time, and if not, is there a way to control it over time? Will the targeted system respond equally well if the targeted substrate changes over time, e.g., upon aging or a secondary disease? How can the therapy be made cardiac-specific or even affected tissue-specific, thus avoiding unnecessary “treatment” of healthy tissue inside or outside the heart? What hurdles, both expected and unexpected, will need to be overcome to successfully translate the therapy from laboratory animals to humans? Will it be possible to organize clinical trials for gene therapy targeting specific ion channel genes, given that the worldwide prevalence of the associated cardiac ion channelopathies may be (too) low?

If each of these “(bio)medical” issues has been successfully addressed, there is still one major non-biomedical issue, which is the economic feasibility of the gene therapy of interest. If, as in the case of SQT3, there are only a few patients worldwide (see Offerhaus et al. [62] and Mariani et al. [63] for recent comprehensive reviews of the prevalence of cardiac ion channelopathies), and the allele-specific silencing requires the development of specific candidate shRNAs and comparison of their ability to specifically target the mutant allele of interest, with minimal affinity for the wild-type gene, this is not the way to go. However, SQT3 patients may benefit from the development of more generic shRNAs targeting common *KCNJ2* variants to silence the mutant *KCNJ2* gene, as in the case of the *KCNQ1* gene [40], which is an approach that is not limited to a single mutation or a single syndrome.

Our simulations were restricted to a 60% suppression of the mutant allele without replacement. In the case of a gain of function, with a substantial remaining I_K1_ amplitude, the replacement is less relevant unless the mutant allele can be knocked down almost completely. The 60% suppression used in our simulations was based on the experimental observations of Cócera-Ortega et al. [40]. However, we could have used a higher percentage, given the knock-down by ≈80% reported by Bains et al. [38], thus reaching a higher functional effect of the suppression.

### 3.5. Limitations

It should be noted that the present study relies entirely on in silico experiments, which had to be based on a limited amount of experimental data, mostly obtained in Kir2.1 expression systems at room temperature (see Section 3.2). In our simulations, we were confronted with a strong model dependence, at least quantitatively, on the results obtained. Apparently, the model dependence of the SQT3 mutation-induced shortening of the action potential duration is largely, but not entirely, due to the distinct formulation of I_K1_ in the two models (see Section 3.3). Further in vitro and in vivo experiments are required to confirm and quantify the computationally observed alleviations.

It seems appealing to obtain hiPSC-CMs from SQT3 patients for in vitro experiments. However, such experiments strongly rely on the maturity of the electrophysiological phenotype of hiPSC-CMs, in particular with respect to the functional expression of I_K1_ channels. In contrast to other ion channels, I_K1_ channels are expressed at significantly lower levels in hiPSC-CMs than in native cardiomyocytes or are even functionally absent [64,65,66]. This strongly reduces the usefulness of hiPSC-CMs for in vitro experiments on SQT3. An alternative would be to use the dynamic clamp technique to express a synthetic WT or mutant I_K1_ in hiPSC-CMs from a control cell line [67], as in the dynamic clamp experiments on guinea pig ventricular myocytes carried out by Du et al. [35]. However, the outcome of such experiments depends largely on the validity of the I_K1_ equations applied, which in turn depends on the extent to which the experimental data from which they are derived are representative of I_K1_ in WT or mutant cardiomyocytes. The most appealing but also complex approach is, therefore, the construction of a transgenic animal model with SQT3, using animals with a human-like ventricular action potential, such as the transgenic rabbit model for type 1 long QT syndrome that was recently developed by Bains et al. [39].

## 4. Materials and Methods

### 4.1. Ventricular Cell Models

The electrophysiology of an isolated human ventricular cardiomyocyte was simulated using the ToR–ORd and BPS2020 models [42,43]. For the ToR–ORd model, we used the CellML code [68,69] that was made publicly available by the developers of the model on the GitHub platform (https://github.com/jtmff/torord; accessed on 26 August 2024). We selected the updated version, termed ToR–ORd–dynCl, with a dynamic representation of the intracellular chloride concentration, which behaves very similarly to the original ToR–ORd model but with higher stability over long simulations [70].

For the BPS2020 model, we used the CellML code that the developers of the model made publicly available on the website of the MCBeng community of researchers in the field of Molecular and Cellular Bioengineering (https://www.mcbeng.it/en/; accessed on 26 August 2024) through a link to a specific page of the CellML Model Repository (https://models.cellml.org/workspace/5fd; accessed on 26 August 2024). We have carefully checked this CellML code against each of the more recent versions that are also available from the CellML Model Repository [71]. This check did not reveal essential differences in the model equations or parameters.

The CellML code of the models was edited and run in version 0.9.31.1409 of the Windows-based Cellular Open Resource (COR) environment [72]. All simulations were run for a simulated period of 2 min, which appeared long enough to achieve steady-state behavior under each simulated condition. The data analyzed are from the final ten seconds of this 2 min period. Action potentials (APs) were elicited with a 1 ms, ≈2× threshold stimulus. Action potential duration (APD) and diastolic interval (DI) were defined as the durations that the membrane potential was positive to −72 mV and negative to −72 mV, respectively [73].

### 4.2. Incorporation of the SQT3 Mutations into the BPS2020 Model

**Figure 11:**
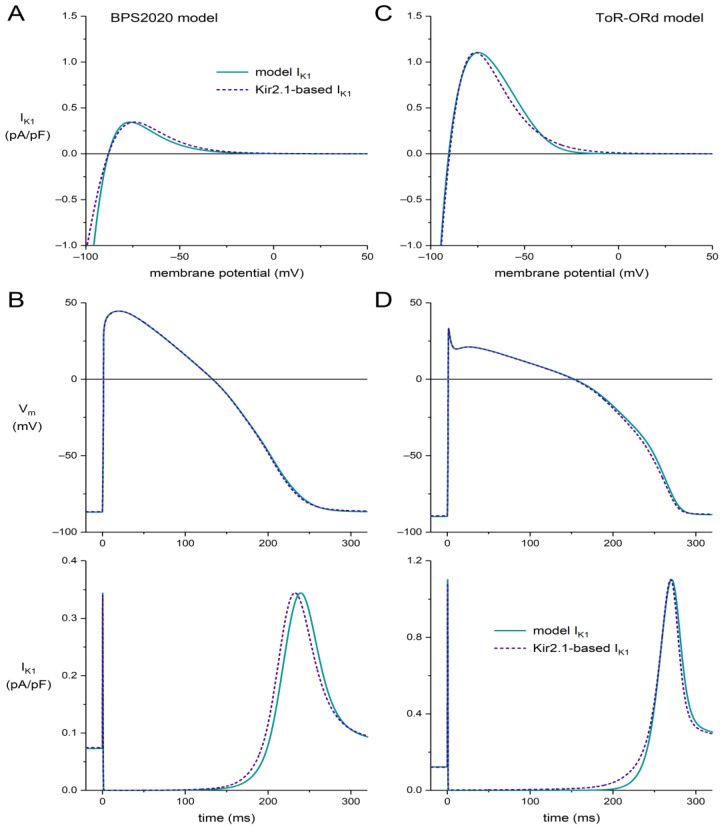
Effect of replacing the original I_K1_ formulations of the BPS2020 and ToR–ORd models with Kir2.1-based formulations. (**A**) Current–voltage relationship of the original I_K1_ of the BPS2020 model and the Kir2.1-based one used in our simulations. (**B**) V_m_ and I_K1_ of the BPS2020 model during 1 Hz stimulation. (**C**) Current–voltage relationship of the original I_K1_ of the ToR–ORd model and the Kir2.1-based one used in our simulations. (**D**) V_m_ and I_K1_ of the ToR–ORd model during 1 Hz stimulation.

In the default BPS2020 model, which represents an endocardial ventricular cardiomyocyte, the I_K1_ formulation was taken from the ORd model but with a +8 mV shift in the current equations to compensate for the difference in the extracellular potassium concentration between the in vitro data used by O’Hara et al. [41] (4.0 mM) and the BPS2020 model (5.4 mM). Also, the maximum conductance of I_K1_ was decreased by 29%, and the steady-state rectification slope was increased by 9% in the “automatic optimization” procedure, in which these two parameters were part of the “cost function” [42]. To be able to directly incorporate the effects of the D172N and E299V SQT3 mutations (see below), we replaced this BPS2020 current–voltage relationship with the “control I_K1_” presented by Deo et al. [19], which is based on in vitro measurements of the current flowing through wild-type Kir2.1 channels expressed in HEK cells, but scaled down by a factor of 0.43751 to retain the maximum outward amplitude of 0.34459 pA/pF of the native I_K1_ of the BPS2020 model (Figure 3A, blue line). Thus, we obtained the following:

I_K1_ = 0.1082·(V_m_ − E_K_)/{0.86426 + exp[0.09014·(V_m_ − E_K_)]},

in which I_K1_ is expressed in pA/pF and V_m_ in mV, and E_K_ is the Nernst potential for K^+^ as reversal potential for I_K1_. Deo et al. [19] had added a small minus 0.06519 shift along the current axis, which appeared to be included because of a leak current. However, in our computations, we have left out this shift to ensure that the simulated I_K1_ is a pure potassium current with E_K_ as its reversal potential. Figure 11A shows the current–voltage relationship of I_K1_ that we introduced compared to the original one of the BPS2020 model, which in turn is based on that of the ORd model (as set out above), which was developed at a time when the in vitro data by Deo et al. [19] were not yet available. The two current–voltage relationships are only slightly different, and the effect of this difference on the action potential of the BPS2020 model is negligible (Figure 11B). To obtain the equation for I_K1_ in the midmyocardial and the epicardial versions of the model, the maximum outward amplitude of the endocardial I_K1_ was scaled by 1.3 and 1.2, respectively.

Now that the wild-type I_K1_ had been based on the in vitro experiments by Deo et al. [19], the equations for the heterozygous WT/D172N, homozygous D172N, heterozygous WT/E299V, and homozygous E299V Kir2.1 currents could all be based on the experiments on these currents that were also carried out by Deo et al. [19]. To this end, we used the equations that Deo et al. [19] fitted to their experimental data. However, the maximum outward amplitudes of these four currents were all scaled to the maximum outward amplitude of the wild-type Kir2.1 current that Deo et al. [19] measured under identical experimental conditions, which we had implemented in the BPS2020 model with a scaling factor of 0.43751 to obtain the original maximum outward amplitude of 0.34459 pA/pF. Accordingly, the heterozygous WT/D172N current was simulated by

I_K1_ = 0.55327·(V_m_ − E_K_)/{4.0041 + exp[0.0841·(V_m_ − E_K_)]},

and the homozygous D172N current by

I_K1_ = 3.3866·(V_m_ − E_K_)/{21.5492 + exp[0.1052·(V_m_ − E_K_)]},

in both cases leaving out the small shift along the current axis that Deo et al. [19] included to compensate for a leak current in order to retain E_K_ as the reversal potential. This reversal potential was −88 mV in our simulations with the BPS2020 model, based on the model’s intracellular and extracellular K^+^ concentrations of 145 and 5.4 mM, respectively. Of note, E_K_ was set to the round value of −90 mV in the current–voltage relationships of Figure 3C–F, as an intermediate between the two slightly different E_K_ values of the BPS2020 and ToR–ORd models.

In a similar way, we arrived at

I_K1_ = 0.043754·(V_m_ − E_K_)/{0.04092 + exp[0.01732·(V_m_ − E_K_ − 2.4)]}

as the equation for the heterozygous WT/E299V current that we used in our simulations with the BPS2020 model. Because Deo et al. [19] originally fitted their homozygous E299V data in a somewhat different way, using a second-order polynomial, we kept their equation to construct Figure 3D, but with a 1.25 mV shift along the voltage axis to arrive at the −90 mV reversal potential used in Figure 3C–F. Accordingly, the homozygous E299V current was simulated by

I_K1_ = 0.43751·[0.06634·(V_m_ − E_K_ + 7.75) − 2.44009·10^−4^·(V_m_ − E_K_ + 1.25)^2^ − 0.51383].

Simulations with the BPS2020 model were run with the heterozygous WT/D172N and WT/E299V currents only, considering that each of the four SQT3 mutations is heterozygous in the clinic. As a consequence, the above equations for the homozygous D172N and E299V currents were only used for Figure 3C,D.

As briefly mentioned in Section 2.1, we used a similar approach, albeit with a third-order polynomial, to fit the experimental data that Hattori et al. [20] obtained on the M301K mutation. The heterozygous I_K1_ shown in their Figure 3B [20] could be well fitted (r^2^ > 0.99) with

I_K1_ = 0.8897 + 3.3933·10^−3^·V_m_ − 5.0165·10^−5^·V_m_^2^ + 2.4534·10^−7^·V_m_^3^.

In this equation, I_K1_ was normalized to its maximum outward amplitude, and V_m_ denotes the membrane potential in mV, which can be shifted along the voltage axis of the current–voltage relationship to obtain the correct I_K1_ reversal potential (i.e., the Nernst potential for K^+^). This reversal potential was −90 mV using the above equation. In Figure 3E, the amplitude of the heterozygous I_K1_ was scaled to the wild-type I_K1_ that was also presented by Hattori et al. [20] in their Figure 3B. In our simulations with the BPS2020 model, the above fit was ultimately scaled by a factor of 1.1945 and shifted along the voltage axis by 2 mV to obtain the I_K1_ reversal potential of −88 mV of the BPS2020 model. Because, as mentioned in Section 2.1, Hattori et al. [20] recorded no functional I_K1_ in the homozygous case, the homozygous M301K I_K1_ was set to zero in Figure 3E.

Finally, the heterozygous WT/K346T and homozygous K346T current–voltage relationships were obtained by scaling the wild-type I_K1_ by 19% and 40%, respectively, as observed experimentally, as already mentioned in Section 2.1, in accordance with the experimental observations presented in Table 2.

### 4.3. Incorporation of the SQT3 Mutations into the ToR–ORd Model

As with the BPS2020 model, the exact formulation of I_K1_ in the development of the ToR–ORd model resulted from a calibration procedure. Specifically, “the ToR–ORd model was calibrated to manifest depolarization of resting membrane potential in response to an I_K1_ block, based on evidence in a range of studies summarized in Dhamoon and Jalife (2005)” [43], referring to the study by Dhamoon and Jalife from 2005 [74]. As noted by Tomek et al. [43], the original ORd model “failed to fulfill the depolarizing effect of I_K1_ block”. In the ToR–ORd model, the I_K1_ formulation of the original ORd model was replaced by the formulation from a simulation study by Carro et al. [75], which was slightly adapted by Tomek et al. [43] to account for the difference in the extracellular potassium (5.0 vs. 5.4 mM). The resulting I_K1_ showed a maximum outward amplitude of 1.1031 pA/pF, as illustrated in Figure 3B, which amounted to 0.34459 pA/pF in the BPS2020 model (see Section 4.2).

To incorporate the SQT3 mutations into the ToR–ORd model, we used the same approach as with the BPS2020 model. However, all I_K1_ amplitudes were scaled up by a factor of 3.2012 to account for the larger maximum outward amplitude of I_K1_ in the ToR–ORd model, as illustrated in Figure 3A,B. Also, each of the equations was used with a reversal potential of −90 mV to account for the slightly more negative E_K_ resulting from the slight differences in intracellular and extracellular K^+^ concentrations between the two models. Figure 11C shows the current–voltage relationship of the wild-type I_K1_ that we introduced compared to the original one of the ToR–ORd model. As in the case of the BPS2020 model, the two current–voltage relationships are only slightly different, and the effect of this difference on the action potential is negligible (Figure 11D). As for the BPS2020 model, the midmyocardial and the epicardial versions of the equation for I_K1_ were obtained from the default endocardial I_K1_ by scaling its maximum outward amplitude by 1.3 and 1.2, respectively.

## Data Availability

The raw data supporting the conclusions of this manuscript will be made available by the author, without undue reservation, to any qualified researcher.
